# Effect of definitive hypo-fractionated radiotherapy concurrent with weekly cisplatin in locally advanced squamous cell carcinoma of the head and neck

**DOI:** 10.25122/jml-2023-0003

**Published:** 2023-05

**Authors:** Nora Abdelhafiz, Doaa Mahmoud, Mohamed Gad, Hoda Essa, Aiat Morsy

**Affiliations:** 1. Department of Radiotherapy and Nuclear Medicine, South Egypt Cancer Institute, Assiut University, Assiut, Egypt.; 2. Department of Clinical Oncology, Faculty of Medicine, Assiut University, Assiut, Egypt.; 3. Department of Clinical Oncology, Saudi German Hospital Aseer, Khamis Mushait, Saudi Arabia; 4. Department of Otorhinolaryngology, Head and Neck Surgery, Faculty of Medicine, Assiut University, Assiut, Egypt

**Keywords:** Cisplatin, hypo-fractionated radiotherapy, head and neck carcinoma, survival, toxicity

## Abstract

To mitigate the risk of COVID-19 infection in cancer patients, it is recommended to utilize hypo-fractionated treatment schedules that aim to minimize the overall duration of treatment. In this study, we aimed to determine whether hypo-fractionated intensity-modulated radiotherapy (hypo-IMRT) with concurrent chemotherapy was practical, effective, and could achieve acceptable tumor control rates for squamous cell carcinoma of the head and neck (SCCHN). We enrolled 62 patients with high-risk stage II, stage III, and IVA SCCHN who received hypo-IMRT (62.5 Gy in 25 fractions over 5 weeks 2.5Gy/fraction with weekly cisplatin 40 mg/m^2^). Our primary endpoint was to assess acute toxicity, while our secondary endpoints were late toxicity, loco-regional control, disease-free survival, and overall survival. The percentages of grade 3 acute pain, dermatitis, mucositis, and dysphagia were 71%, 19.4%, 72.6%, and 41.9%, respectively. The rates of late xerostomia, dysphagia, dental complications, grade 3 pain, and grade 3 weight loss were 72.6%, 62.9%, 27.4%, 4.8%, and 4.3%, respectively. At a median follow-up time of 24 months, 2-year loco-regional control and overall survival were 87.1% and 83.9%, respectively. Disease-free survival was 100%, 89.5%, and 69% in stages II, III, and IV%, respectively, with a significant p-value of 0.024. This regimen was effective and relatively safe, with acceptable and tolerable acute and late toxicity. Given the reduced need for hospital visits, hypo-fractionated schedules may represent an alternative treatment during the COVID-19 outbreak.

## INTRODUCTION

Head and neck cancer is considered the 6th most common cancer worldwide, with 890,000 new cases and 450,000 deaths in 2018 [1]. Among the various histological types, squamous cell carcinoma (SCC) is the most frequently diagnosed form of head and neck cancer [2]. The projected incidence of SCC in head and neck cancer (SCCHN) is expected to increase by 30% by 2030, translating to approximately one million new cases annually [3]. According to GLOBOCAN 2012 estimates, age-standardized incidence rates per 100,000 per annum were 2.6 for males and 1.8 for females. The age-standardized mortality rates for oral cavity and oropharyngeal cancer were 1.1 and 0.7 for males and females, respectively. Notably, the incidence and mortality rates are predicted to double by 2030, especially in Egypt, Iran, Morocco, Sudan, and Turkey, which is twice the projected worldwide rate [4]. Hospital-based studies in Egypt have indicated that SCCHN accounts for roughly 20% of all malignancies. In Egypt, from 1999 to 2006, the incidence of SCCHN was higher in males (476,000) than in females (273,000) [5, 6].

The standard treatment for SCCHN involves a multimodality approach combining surgery, radiotherapy (RT), and chemotherapy (CTH). Clinical trials have shown that utilizing altered fractionated RT with CTH, as opposed to conventional fractionated RT (CFRT), leads to substantial improvements in loco-regional tumor control (LRC) [7,8]. CFRT is delivered at 1.8 or 2.0 Gy per fraction, but different alterations of RT are called altered fractionated RT, such as hyper-fractionated RT, accelerated-fractionated RT, and hypo-fractionated RT (hypo-RT) [9].

Altered fractionated radiotherapy (RT) schedules have been shown to reduce the tumor repopulation effect and improve the therapeutic ratio between tumor cell killing and normal tissue damage, as evidenced by randomized controlled trials comparing them with conventional fractionated RT (CFRT) with chemotherapy (CTH) for the treatment of head and neck cancer [10-13]. Hypo-fractionated RT (hypo-RT) is a type of altered fractionated schedule that uses a smaller number of larger fractions (>2 Gy per fraction) for shortening the overall treatment time compared to CFRT [6]. This schedule is particularly beneficial for cancer patients, who are at a higher risk of COVID-19 infection due to frequent hospital visits. However, a higher dose per fraction might increase the incidence of late complications [14,15]. The aim of our study was to assess the efficacy and safety of hypo-fractionated intensity-modulated RT (hypo-IMRT) with 62.5 Gy in 25 daily fractions over 5 weeks (2.5 Gy per fraction size with weekly cisplatin 40 mg/m2) for patients with high-risk stage II, III, and IV squamous cell carcinoma of the head and neck (SCCHN). The primary endpoint was to evaluate acute toxicity, while the secondary endpoints included the assessment of late toxicity, loco-regional control (LRC), disease-free survival (DFS), and overall survival (OS).

## MATERIAL AND METHODS

In our prospective phase I/II study, 62 patients were enrolled at the South Egypt Cancer Institute in Assiut, Egypt, between 2018 and 2021. Patients were eligible if they were classified into high-risk stage II (T2N0 that was not amenable for laryngeal preservation surgery, excluding glottic laryngeal disease), stage III (T1-3N1 or T3N0), or stage IVA (T1-4N2) oropharyngeal (32.3%), laryngeal (35.5%) or hypopharyngeal SCC (32.3%), according to the American Joint Committee on Cancer (AJCC) staging classification 2018 (8th edition). Their ages were between 30 and 78 years, with an Eastern Cooperative Oncology Group (ECOG) performance status of ≤2 and adequate organ function. Exclusion criteria included previous treatment with CTH or RT, previous surgical curative resection of the primary tumor, any relative contraindication to RT, active severe infection, active concomitant malignancy, preexisting motor or sensory neurotoxicity > Common Terminology Criteria of Adverse Events (CTCAE) grade 2, pregnancy or lactation.

### Radiotherapy and chemotherapy protocol

Patients received hypo-IMRT with weekly cisplatin at 40 mg/m^2^. All patients underwent simulation with head immobilization using a thermoplastic head and neck mask, and contrast-enhanced CT images were obtained for treatment planning at 2.5 mm intervals from the vertex to below the carina. CT images were transmitted to the Monaco Treatment Planning System. The treatment was delivered using a linear accelerator with a 6-megavoltage photon beam through an inversely planned IMRT and a 7-field beam arrangement. The gross tumor volume (GTV) in this study referred to the primary tumor and the involved lymph nodes, as determined by clinical and radiological investigations. Lymph nodes were detected as those were more than 10 mm in short axis diameter. The clinical target volume (CTV) was the GTV and subsites of high-risk microscopic disease. Three CTVs were delineated: CTV1, which included a 0.5 cm margin around the GTV (excluding air, fascia, and bone); CTV2, which included the remainder of the involved subsite, lymph nodal levels, and the uninvolved high-risk first echelon nodal regions; and CTV3, which encompassed the low-risk nodal levels of microscopic disease spread. Three planning target volumes (PTV1, PTV2, and PTV3) encompassing CTV1, CTV2, and CTV3 with an expansion of 5 mm margin, respectively. Organs at risk (OAR) were also outlined on the axial images. The radiation doses to PTV1, PTV2, and PTV3 were 62.5 Gy, 55 Gy, and 50 Gy over 5 weeks, respectively, prescribed to the mean of the PTV. The minimum and maximum doses administered to the PTV were maintained within the range of 95% to 107% of the prescribed dose. Treatment verification was made using an electronic portable image device (EPID) during the first 3 treatment fractions and then weekly after that. The concurrent CTH was weekly cisplatin 40 mg/m^2^ with proper good hydration and premedication with IV dexamethasone and diphenhydramine

### Pretreatment evaluation

Pretreatment evaluation of the tumor was measured by CT, MRI, and direct endoscopy. We completed a medical history and physical examination for all our patients to ensure their performance status was ≤2. All patients underwent a dental evaluation before RT. During the hypo-RT protocol, complete laboratory and creatinine clearance was requested weekly before the CTH regimen. We used carboplatin AUC 2 instead of cisplatin when the creatinine clearance was less than 60 ml/min.

### Toxicity and tumor response evaluation

Patients were monitored for acute toxicity weekly during treatment and monthly for 6 months after finishing the treatment. Toxicity was assessed based on the CTCAE (version 5), which describes an adverse event (AE) as any unfavorable and unintended sign (including an abnormal laboratory finding), symptom, or disease temporally associated with the use of medical treatment, clinical examination, and measurement of weight. For patients with grade 3 toxicities, we interrupted RT until toxicity resolved to CTCAE grade 2. Supportive medications, such as analgesics and topical moisturizers, were prescribed to manage symptoms. Any delays in RT were carefully compensated for to ensure that the overall treatment time remained within the planned timeframe. Late toxicity was assessed at the 6th month and then monthly until 24 months after completion of treatment using physician- and patient-recorded questionnaires. Tumor response was evaluated 6-8 weeks post-treatment completion using clinical examination, as well as head and neck MRI or CT scans, based on the Response Evaluation Criteria in Solid Tumors (RECIST) criteria. Endoscopy was performed 8-12 weeks after the end of concurrent chemoradiation (CCRT) for pathological confirmation of the response

### Statistical methods

The sample size was calculated using the G-power 3.1 program, and all statistical analyses were conducted using SPSS version 22 (Statistical Package for the Social Science, SPSS Inc., Chicago, IL, USA). Descriptive statistics were reported as mean ± standard deviation (±SD), median and range (for non-normally distributed data), frequencies (number of cases), and relative frequencies (percentages) as appropriate. As the expected frequency was less than 5, the exact test was used instead of the chi-square (χ2) test for comparing categorical data. A p-value of 0.05 was considered statistically significant for all two-tailed tests. The Kaplan–Meier method was used to estimate OS and DFS.

## RESULTS

### Patient characteristics

A total of 62 patients were enrolled in this study from 2018 to 2021. The majority of patients were males (n=52, 83.9%), and the remaining 10 (16.1%) were females. Among them, 22 were current smokers. Patients had histologically or cytologically confirmed oropharyngeal (32.3%), laryngeal (35.5%), or hypopharyngeal SCC (32.3%), with high-risk stage II (22.6%), stage III (30.6%) and stage IV (T1-4N2) (46.8%). HPV status was not evaluated in this study. The baseline and pathological characteristics of the participants are shown in Table 1.

**Table 1.** Column I. Baseline and pathological characteristics of the participants (n=62)

Variable name		
**Age (years)**		
Mean ± SD	59.52 ± 11.89	
Median (range)	60.0 (30 – 78)	
**Variable name**	**N**	**(%)**
**Sex**		
Male	52	(83.9)
Female	10	(16.1)
**Smoking Status**		
Current	22	(35.5)
Former for more than 1 year	17	(27.4)
Former less than 1 year	10	(16.1)
Never	13	(21.0)
**ECOG Performance** **Status “1”**	62	(100.0)
**Tumor site**		
Oropharynx	20	(32.3)
Tonsil	1	(5.0)
Base of the tongue	5	(25.0)
Soft palate	4	(20.0)
Pharyngeal wall	10	(50.0)
Hypopharynx	20	(32.3)
Pyriform fossa	2	(10.0)
Pharyngeal wall	12	(60.0)
Post-cricoid	6	(30.0)
Larynx	22	(35.5)
Supraglottic	4	(18.2)
Glottic	2	(9.1)
Subglottic	0	(0.0)
More than one region/Trans glottic	16	(72.7)
**Pathology**		
Squamous cell carcinoma	62	(100.0)
**Differentiation**		
Well to moderate	8	(12.9)
Moderate	25	(40.3)
Poor	29	(46.8)
**TNM (T)**		
T1	0	(0.0)
T2	33	(53.2)
T3	20	(32.3)
T3	9	(14.5)

**Table 1.** Column II. Baseline and pathological characteristics of the participants (n=62)

Variable name	N	(%)
**TNM (M)**		
M0	62	(100.0)
**Stage**		
Stage II	14	(22.6)
Stage III	19	(30.6)
Stage IV	29	(46.8)

SD: standard deviation; ECOG: Eastern Cooperative Oncology Group; TNM: tumor (T), nodes (N), and metastases (M). Quantitative data are presented as the mean ± SD and median (range), and qualitative data are presented as numbers (percentages).

### Acute toxicity

**Figure 1.**
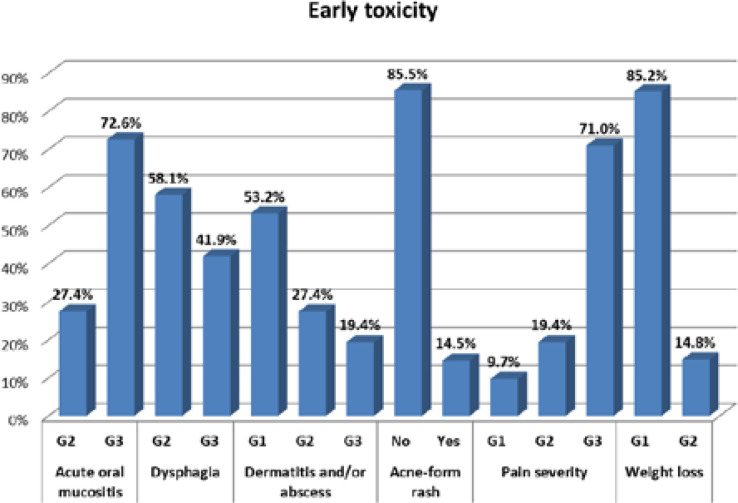
Distribution of early toxicity by grade among patients

Forty-five patients (72.6%) had grade 3 mucositis, 2 of whom needed hospitalization and were compelled to interrupt RT. Twenty-six patients (41.9%) experienced grade 3 dysphagia, and 12 patients (19.4%) developed grade 3 dermatitis. Two patients had an abscess and pus discharge that required interrupting RT and were hospitalized to receive intravenous antibiotics. An additional 2 patients were forced to interrupt RT due to the device being out of function, and the median number of fractions in RT interruption was 3. The RT protocol was completed by 58 patients. 14.5% of our patients experienced acne-form rash, and 71% had grade 3 pain that needed strong analgesics. A total of 14.8% had grade 2 weight loss (more than 10% of their baseline weight) and received nutritional support in the form of oral supplements. The patient-reported early toxicity and the distribution of the percentage of early toxicity of participants by grade are shown in Figure 1.

### Patient-reported late toxicity

The incidence of taste alteration, xerostomia (dry mouth), voice alteration, dysphagia, dental complications, and grade 3 weight loss was 83.9%, 72.6%, 67.7%, 62.9%, 27.4%, and 4.3%, respectively. The incidences of osteoradionecrosis, chronic aspiration, skin fibrosis, and grade 3 late pain were lower. The patient-reported late toxicity that started 6 months after treatment completion is shown in Table 2.

**Table 2.** Patient-reported late toxicity (n=62) (started 6 months after treatment completion)

Late toxicity	N	(%)
**Late Pain severity**	**29**	**(46.8)**
G1	22	(35.5)
G2	4	(6.5)
G3	3	(4.8)
**Late Pain site**	**29**	**(46.8)**
Mouth	13	(44.8)
Throat	27	(93.1)
Jaw	11	(37.9)
Neck	10	(34.5)
Skin	3	(10.3)
Ear	8	(27.6)
**Weight loss**	**47**	**(75.8)**
G1	27	(57.4)
G2	18	(38.3)
G3	2	(4.3)
**Anorexia**	**15**	**(24.2)**
**Dysphagia**	**39**	**(62.9)**
**Skin fibrosis**	**30**	**(48.4)**
G1	24	(80.0)
G2	6	(20.0)
**Dental complication**	**17**	**(27.4)**
**Xerostomia**	**45**	**(72.6)**
**Taste alteration**	**52**	**(83.9)**
**Voice alteration**	**42**	**(67.7)**
**Chronic aspiration**	**4**	**(6.5)**
**Osteoradionecrosis**	**2**	**(3.2)**
**Otitis**	**8**	**(12.9)**

G: grade. Qualitative data are presented as numbers (percentages).

### Treatment evaluation and follow-up outcomes

Treatment response was assessed over 24 months of follow-up. Fifty-six patients reached CR, and 3 achieved partial response (PR), while 3 patients did not respond to treatment and showed tumor progression in the initial evaluation 2 months after the treatment. Fifty-four patients showed 2y-LRC, while 5 patients developed recurrence, as shown in Tables 3 and 4.

**Table 3.** Outcome of the studied cohort (n=62)

Outcome	N	(%)
**Treatment response**		
Complete response	56	(90.3)
Partial response	3	(4.8)
**2-year loco-regional failure**		
Progression	3	(4.8)
Recurrence	5	(8.1)
**2-year LRC**		
No	8	(12.9)
Yes	54	(87.1)
**2-year distant control**		
Nonmetastatic	52	(83.9)
Metastatic	10	(16.1)
**Death status**		
Survived 2 years	52	(83.9)
Died	10	(16.1)
**Follow-up (months)**		
Mean ± SD	23.19 ± 3.25	
Median (range)	24 (2 – 24)	

LRC: loco-regional control; SD: standard deviation. Quantitative data are presented as the mean ± SD and median (range), and qualitative data are presented as numbers (percentages).

**Table 4.** Patients outcomes according to the tumor stage (n=62)

Response	Stage II (n =14)		Stage III (n =19)		Stage IV (n =29)		P value
Complete response	14	(100.0)	19	(100.0)	23	(79.3)	0.039*
Partial response	0	(0.0)	0	(0.0)	3	(10.3)	0.311
Progression	0	(0.0)	0	(0.0)	3	(10.3)	0.311
Recurrence	0	(0.0)	2	(10.5)	3	(10.3)	0.591
2-year LRC	14	(100.0)	18	(94.7)	22	(75.9)	0.053

LRC: loco-regional control. Qualitative data are presented as numbers (percentages). Significance was defined by p < 0.05.

Ten patients developed metastases and continued to receive CTH, 2 of whom showed tumor progression, 5 had loco-regional recurrences, and 3 had a PR. Ten patients died within a median of 13 months because of tumor progression and metastases (8 patients) and non-cancer-related deaths (2 patients).

**Figure 2.**
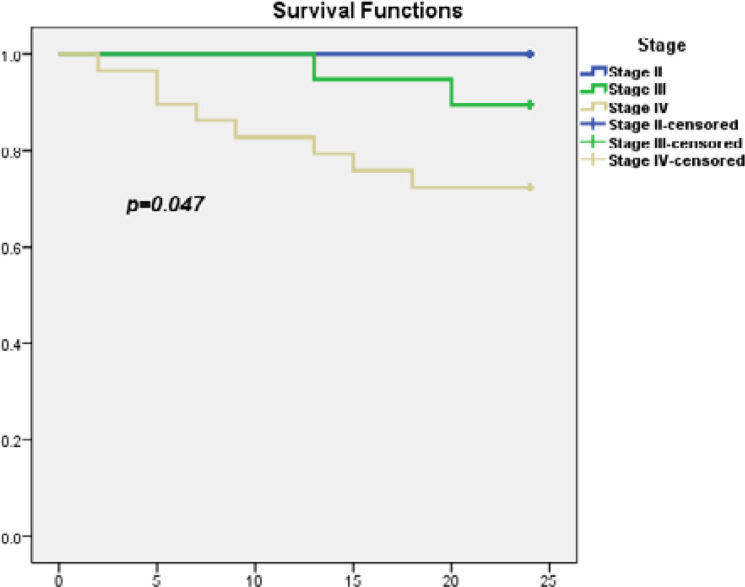
Overall survival of the cohort according to the tumor stage

The evaluation of treatment response was significantly associated with the tumor stage. Detailed information regarding the outcome of the studied cohort based on tumor stage can be found in Table 4. The overall survival (OS) of the cohort is presented at various time points in Table 5, with further stratification according to tumor stage. Statistical significance in OS is demonstrated in Table 6 and Figure 2. The DFS of the studied cohort at different time points is shown in Table 5 and detailed according to tumor stage in Table 6, showing statistical significance.

**Table 5.** Overall survival and disease-free survival of the cohort (n=62)

Death event	OS (Estimate ± SE)
At 2 months	98.4 ± 1.6%
At 5 months	95.2 ± 2.7%
At 7 months	93.5 ± 3.1%
At 9 months	91.9 ± 3.5%
At 13 months	88.7 ± 4.0%
At 15 months	87.1 ± 4.3%
At 18 months	85.5 ± 4.5%
At 20 months	83.9 ± 4.7%
At 24 months	83.9 ± 4.7%
Number of events	10/62
**Disease event**	**DFS (Estimate ± SE)**
At 1 month	98.4 ± 1.6%
At 10 months	96.8 ± 2.2%
At 14 months	93.5 ± 3.1%
At 15 months	91.9 ± 3.5%
At 16 months	90.3 ± 3.9%
At 20 months	85.5 ± 4.5%
At 24 months	82.3 ± 4.9%
Number of events	11/62

OS: overall survival; DFS: disease-free survival; SE: standard error

**Table 6.** Overall survival and disease-free survival of the cohort according to tumor stage (n=62)

OS	Estimate ± SE			P value
	Stage II	Stage III	Stage IV	
At 1 year	100.0 ± 0.0%	100.0 ± 5.1%	82.8 ± 7.0%	0.047*
At 2 years	100.0 ± 0.0%	89.5 ± 7.0%	72.4 ± 8.3%	
N of event	0/14	2/19	8/29	
**DFS**	**Estimate ± SE**			**P value**
	Stage II	Stage III	Stage IV	
At 1 year	100.0 ± 0.0%	100.0 ± 5.1%	93.1 ± 4.7%	0.024*
At 2 years	100.0 ± 0.0%	89.5 ± 7.0%	69.0 ± 8.6%	
N of event	0/14	2/19	9/29	

OS: overall survival; DFS: disease-free survival

## DISCUSSION

The use of CCRT with CFRT is the standard strategy in the management of locally advanced SCCHN. The concurrent use of CTH (3-weekly high-dose cisplatin) is considered the standard of care in managing locoregionally advanced SCCHN [16-18]. However, Medina *et al*. and Beckmann *et al*. showed improvement in LRC and/or survival with weekly cisplatin regimens of 40–60 mg for 6–7 weeks [19,20].

The COVID-19 pandemic may necessitate shorter RT fractions to reduce patient visits. Hypo-RT schedules with larger doses per fraction and fewer fractions had the theoretical advantage of improving the LRC by increasing the cell-killing effect and decreasing the effect of rapid tumor cell repopulation by decreasing the overall RT treatment time frame. However, hypo-RT may result in an increased incidence of acute and late toxicities [21,22]. Current studies are scarce for hypo-RT doses, particularly for once-daily therapy. Therefore, our study investigated the tumor response and toxicity in SCCHN using our hypo-IMRT treatment dose for locally advanced SCCHN.

The main objective of our study was to assess acute toxicity as the primary endpoint. Our radiation schedule was at the upper limit of acceptability, but we did not observe any dose-limiting grade 4 toxicities. Among the patients, 72.6% experienced grade 3 mucositis, and out of 6 patients, 2 (33.3%) required hospitalization and had to interrupt their RT. Our findings were consistent with those of Thomson *et al*., who reported a similar rate of grade 3 mucositis and hospitalization requirement in their study on intermediate-stage oropharyngeal cancer using a hypo-fractionated RT protocol with cetuximab, compared to our study on stage II, III, and IV oropharyngeal, hypopharyngeal, and laryngeal cancer with weekly cisplatin. Specifically, their study reported a 78% rate of grade 3 mucositis and a 41% hospitalization requirement [23]. Trotti *et al*. reported that severe (grade 3/4) mucositis rates were 34%, 43%, and 57% in patients who received CRT alone, synchronous CTH, or altered fractionation schedules, respectively, in a systematic review of 33 studies involving 6181 patients [24]. Hospitalization was required for 21%, 14%, and 66% of the patients in 3 of these studies. Neeraj *et al*. compared a conventional arm (70 Gy/2 Gy/fraction over 7 weeks) with a hypofractionated arm (55 Gy/2.75 Gy/fraction over 4 weeks), and both arms received weekly cisplatin (40 mg/m^2^). Grade ≥ 2 mucositis was also higher in the hypo-fractionated arm (88% vs. 40% in the conventional arm; p≤0.001) [25].

A percentage of 19.4% of our patients experienced grade 3 dermatitis compared to 30% in a study performed over 20 patients with unresectable stage III & IV using IMRT, which used 55 Gy/20 fractions with concomitant 4 weekly cycles of cisplatin 35 mg/m^2^ [26]. This discrepancy in results was due to a larger fraction size over a shorter overall treatment time (2.75 Gy/fraction over 4 weeks in the Jacinto trial compared to our study, which used 2.5 Gy/fraction over 5 weeks. Additionally, Thomson *et al*. reported an acne-form rash of 8% compared to 14.5% in our patients and grade 3 pain of 81% to 71% in our patients who needed strong analgesics. Similar results of grade 3 dysphagia were 41.9% and 41%, respectively [23].

In an Australian study by OTTY *et al*. that analyzed 102 patients receiving CFRT with weekly cisplatin 40 mg/m2 from 2003 to 2009, the documented rates of mucositis, dysphagia, and grade 3 radiation dermatitis were 21.8%, 12.9%, and 8.9%, respectively [27]. Our result was predicted due to the known biologically equivalent dose (BED) of acute tissue reactions of the mucosa. We can use a linear quadratic equation to calculate the BED and predict the acute toxicity of altered fractionated RT regimens [28]. Fowler *et al*. proved that the tolerance threshold of acute mucosal reactions should not exceed the BED of 59 – 61 Gy_10_ [29]. The equivalent dose for CFRT delivering 70 Gy in 35 daily fractions over 7 weeks was BED acute mucosa 53.1 Gy_10_. Although the corresponding dose for our tested regimen of 62.5 Gy in 25 daily fractions over 5 weeks was BED acute mucosa 58.3 Gy_10_, it did not exceed the tolerable threshold dose. This indicates that our safety from acute toxicities was at the upper limit of acceptable tolerability.

In the Skladowski *et al*. trial that used continuous accelerated irradiation (CAIR) with 2D-RT, patients received 66-72 Gy (74% of patients received 72 Gy) in 2 Gy fractions delivered over 5 weeks. The study showed poorly tolerated toxicities, with significantly increased acute toxicity and subsequent late soft tissue toxicity in 22% of patients, likely due to exceeding the tolerance threshold for acute mucosal reactions of 59-61 Gy_10_ using old 2D techniques [30]. Accelerated RT with a shortening of overall treatment time by 1 or 2 weeks using IMRT appears to be a practical and effective option for managing locally advanced SCCHN [31].

Our study found that 14.8% of patients required nutritional support due to grade 2 weight loss (more than 10% of their baseline weight), higher than the 7% reported by Thomson *et al*. This difference may be due to lower incomes in our study population, which is typical of developing countries [10].

In our study, the treatment completion rate was comparable to that of the Jacinto *et al*. study but with a longer overall treatment time due to the smaller fraction size and longer treatment duration. Meanwhile, the completion rate in the Thomson *et al*. study was slightly lower than that in our study, and a significant proportion of patients experienced treatment delays due to various reasons, including pain, mucositis, facial swelling, and complications related to gastrostomy insertion [23].

The incidence of severe late toxicities (grade 3) in our study was low. The incidence of taste alteration, xerostomia, voice alteration, dysphagia, dental complications, and grade 3 weight loss was 83.9%, 72.6%, 67.7%, 62.9%, 27.4%, and 4.3%, respectively, compared to the study of Thomson *et al*. (78%, 89%, 67%, 70%, 42%, and 4%, respectively) [17]. The incidence of osteoradionecrosis, chronic aspiration, skin fibrosis, and grade 3 late pain was low. These results were in line with the expected outcomes predicted by the BED modeling for late reactions, as the equivalent doses for CFRT receiving 70 Gy in 35 daily fractions over 7 weeks are BED late effects of 116.7 Gy_3_, while the comparable dose received in our protocol was 114.6 Gy_3_.

In the Danish Head and Neck Cancer (DAHANCA)-6 & 7 trial, an accelerated schedule of 66–68 Gy in 33–34, 6 weekly fractions using a conventional technique (BED acute mucosa 55.4–57.0 Gy_10_ and BED late effects 110–113 Gy_3_) improved LRC by 10% at 5 years, with associated increased acute toxicities, but reversible and showed no significant increases in late effects [32].

In contrast, Neeraj *et al*. found that 17 patients (68%) in a conventional arm (70 Gy/2 Gy/fraction/7 weeks) achieved a complete response (CR), and 15 patients (60%) in a hypo-RT arm (55 Gy/2.75 Gy/fraction/4 weeks) had CR. In our study, after a median follow-up of 24 months, the CR rate was 90.3%, with 100% in stages II, III and 79.3% in stage IV (p-value=0.039) [25]. Three patients (4.8%) had PR and developed metastases at an average time of 15 months, and one of these patients subsequently died of the disease. Three (4%) patients had a progressive disease that was not amenable to surgical resection; 1 of them died after 2 months, and the other 2 patients subsequently developed metastases and died at an average time of 16 months. Five (8.1%) patients had a loco-regional recurrence and became metastatic, and the median time to recurrence and metastases was 15 months. The total percentage of metastases and death was 16.1% for each. Thomson *et al*. reported that 4% of their patients had a PR without evidence of disease on subsequent follow-up, 4% had progressive disease, which was not amenable to surgical resection and then died of the disease later, and 8.1% had loco-regional recurrence with an average recurrence time of 18.7 months, who subsequently died.

Sanghera *et al*. reported 2y-LRC (75.4%), 2y-OS (71.6%), and 2y-DFS (68.6%) in a study performed on 81 patients with SCC of the larynx, oropharynx, oral cavity, and hypopharynx who received hypo-RT at a dose of 55 Gy in 20 fractions with concurrent CTH. Detailing their results according to tumor stage, they reported 2y-LRC in stage II (92.9%), stage III (85%), stage IV (64.7%), 2y-OS in stage II (92.9%), stage III (90%), stage IV (57.6%) and 2y-DFS in stage II (92.9%), stage III (80%), stage IV (54.6%), while in our result the 2y-LRC in stage II (100%), stage III (94.7%), stage IV (75.9%), 2y-OS in stage II (100%), stage III (89.5±7.0%), stage IV (72.4±8.3%) and 2y-DFS in stage II (100%), stage III (89.5±7.0%), stage IV (69.0±8.6) [33].

Patients with earlier stages in our study achieved a greater benefit in local control and survival, approaching the results achieved by Sanghera *et al*. However, we observed a slight increase in LRC, DFS, and OS benefits in stage IV due to our higher total dose (62.5 Gy instead of 55 Gy) over a slightly longer duration (5 weeks instead of 4 weeks).

Consistent with previous studies, the tumor stage strongly affected the outcome results. Our findings showed a 2-year OS of 100.0±0.0% for those with stage II and 72.4±8.3% for those with stage IV, with a statistically significant difference (p-value=0.047*).

## CONCLUSION

This regimen was effective and relatively safe with acceptable acute and late toxicity, especially in stages II and III and, to some extent, in stage IV. This approach could represent an alternative during the COVID-19 outbreak due to fewer hospital visits, especially in elderly and comorbid patients, and a lower load on linear accelerator machines. Further studies with larger samples and more advanced techniques are recommended to evaluate the effectiveness of this regimen.
